# Airway M Cells Arise in the Lower Airway Due to RANKL Signaling and Reside in the Bronchiolar Epithelium Associated With iBALT in Murine Models of Respiratory Disease

**DOI:** 10.3389/fimmu.2019.01323

**Published:** 2019-06-11

**Authors:** Shunsuke Kimura, Mami Mutoh, Meri Hisamoto, Hikaru Saito, Shun Takahashi, Takanori Asakura, Makoto Ishii, Yutaka Nakamura, Junichiro Iida, Koji Hase, Toshihiko Iwanaga

**Affiliations:** ^1^Laboratory of Histology and Cytology, Graduate School of Medicine, Hokkaido University, Sapporo, Japan; ^2^Division of Biochemistry, Faculty of Pharmacy, Keio University, Tokyo, Japan; ^3^Department of Orthodontics, Faculty of Dental Medicine, Graduate School of Dental Medicine, Hokkaido University, Sapporo, Japan; ^4^Division of Oral Functional Science, Department of Oral Functional Prosthodontics, Graduate School of Dental Medicine, Hokkaido University, Sapporo, Japan; ^5^School of Medicine, Hokkaido University, Sapporo, Japan; ^6^Division of Pulmonary Medicine, Department of Medicine, Keio University School of Medicine, Tokyo, Japan

**Keywords:** M cells, microfold cells, iBALT, GP2, RANKL, lower airway

## Abstract

Microfold (M) cells residing in the follicle-associated epithelium of mucosa-associated lymphoid tissues are specialized for sampling luminal antigens to initiate mucosal immune responses. In the past decade, glycoprotein 2 (GP2) and Tnfaip2 were identified as reliable markers for M cells in the Peyer's patches of the intestine. Furthermore, RANKL–RANK signaling, as well as the canonical and non-canonical NFκB pathways downstream, is essential for M-cell differentiation from the intestinal stem cells. However, the molecular characterization and differentiation mechanisms of M cells in the lower respiratory tract, where organized lymphoid tissues exist rarely, remain to be fully elucidated. Therefore, this study aimed to explore M cells in the lower respiratory tract in terms of their specific molecular markers, differentiation mechanism, and functions. Immunofluorescence analysis revealed a small number of M cells expressing GP2, Tnfaip2, and RANK is present in the lower respiratory tract of healthy mice. The intraperitoneal administration of RANKL in mice effectively induced M cells, which have a high capacity to take up luminal substrates, in the lower respiratory epithelium. The airway M cells associated with lymphoid follicles were frequently detected in the pathologically induced bronchus-associated lymphoid tissue (iBALT) in the murine models of autoimmune disease as well as pulmonary emphysema. These findings demonstrate that RANKL is a common inducer of M cells in the airway and digestive tracts and that M cells are associated with the respiratory disease. We also established a two-dimensional culture method for airway M cells from the tracheal epithelium in the presence of RANKL successfully. This model may be useful for functional studies of M cells in the sampling of antigens at airway mucosal surfaces.

## Introduction

The mucosal surface of the lower airway is exposed to a vast array of potentially harmful foreign pathogens and antigens derived from inhaled air. This site, comprising the larynx, trachea, and bronchioles, is lined by the pseudostratified epithelium that comprises mainly of ciliated, goblet, club, tuft, and basal cells. The bronchioles continue to divide and give rise to many terminal tubes called alveolar ducts, in which numerous macrophages reside. The goblet cells, ciliated cells, and alveolar macrophages collaborate to keep the airways clean via mucociliary and phagocytic clearance systems (1, 2). In addition, there is a large amount of secretory IgA (sIgA), which plays a major role in mucosal defense, in the airway mucosa (3).

Microfold (M) cells, which are found in the mucosal epithelium associated with lymphoid follicles in the intestine, are one epithelial cell type that specializes in the transport of macromolecules and microorganisms from the intestinal lumen into the subepithelial region via a transepithelial pathway; this process is known as antigen transcytosis (4–6). The luminal antigens are transferred by M cells to immature dendritic cells (iDCs) that accumulate at the subepithelial dome region; subsequently, the iDCs undergo maturation and, in turn, activate antigen-specific naïve T cells. Thus, M-cell-dependent antigen transcytosis may play a key role in the induction of mucosal immune responses, especially in sIgA production, against certain antigens.

In the last decade, studies have identified various unique molecules expressed in the M cells of Peyer's patches. Among them, glycoprotein 2 (GP2) was recognized as a reliable indicator of mature and functional M cells in the intestine (7). GP2 is expressed on the luminal surface of M cells and acts as a receptor that mediates the uptake of type-I-piliated bacteria residing in the gut (8). Tnfaip2 is a cytosolic protein that is expressed in mature and immature M cells (7) and is considered to play a role in intercellular communication among M cells (9–12). The receptor activator of nuclear factor-κB (RANK) and its ligand (RANKL) are indispensable for initiating M-cell differentiation from Lgr5-positive epithelial stem cells (13, 14). RelB and Spi-B, transcription factors downstream of RANKL–RANK signaling, have been shown to be essential for the subsequent differentiation process toward the commitment of intestinal M cells (7, 14–16). More recently, we identified Sox8, a member of the SRY-related HMG-box family, as another transcription factor for the maturation of M cells (17).

Nasopharynx (or nasal)-associated lymphoid tissue (NALT) nestles along both sides of the nasopharyngeal duct and is generally considered to correspond to Waldeyer's ring in humans (18, 19). Similar to Peyer's patches in the intestinal immune system, NALT serves as a site for the induction of mucosal immunity in the upper airway. We previously reported that GP2^+^Tnfaip2^+^ cells on the follicle-associated epithelium of NALT exhibit morphological features typical of M cells, share the ability to take up luminal microbeads, and express additional molecules specific to intestinal M cells, including Spi-B and Ccl9 (20). Furthermore, the differentiation of GP2^+^Tnfaip2^+^ cells of the NALT is regulated by RANKL–RANK signaling. The GP2^+^Tnfaip2^+^ cells in the nasal cavity therefore correspond to intestinal M cells.

In the lower airway, the lymphoid follicles rarely appear in healthy humans and mice. Few studies have suggested the presence of M cells in the lower airway, so the molecular characteristics of M cells at this site have remained unclear. In this study, we investigated the expression profiles of M-cell-related molecules in the lower respiratory tract of mice in healthy and pathological conditions. We also succeeded, for the first time, in generating airway M cells in two-dimensional culture conditions from the murine tracheal epithelial cells.

## Methods

### Mice

Seven- to twelve-week-old BALB/cCrSlc (BALB/c), C57BL/6NCrSlc (C57BL/6N), and Slc:ddY mice were purchased from Japan SLC. Sixteen-weeks female NOD/ShiJcl mice were purchased from CLEA Japan. These mice were maintained under conventional conditions. For elastase-induced emphysema model and cigarette smoke exposure, female WT C57BL/6JJcl (C57BL6J) mice (8–10 weeks old) were purchased from CLEA Japan.

All experiments using animals were performed under protocols following the Guidelines for Animal Experimentation, of Hokkaido University Graduate School of Medicine (Protocols No. 15-0139 and No. 18-0034) and the Animal Use Committee at the Keio University School of Medicine approved all animal experiments (protocols No. 12109 and No. 12110).

### Immunohistochemistry and Immunofluorescence Analysis for the Trachea

Deeply anesthetized mice were perfused via the aorta with physiological saline followed by 4% paraformaldehyde (PFA), pH 7.4. The trachea or the lung were removed and immersed in same fixative for an additional 24 h. For frozen section, the specimen were dipped in 30% sucrose solution at 4°C overnight, embedded in optimum cutting temperature (O.C.T) compound, and quickly frozen in liquid nitrogen. Frozen 10-μm-thick sections were mounted on MAS-coated slide glass and air-dried (Matsunami glass).

For paraffin sections, the specimen were dehydrated by immersing an increasing concentration of alcohol and subsequently cleared in xylene before mounting in paraffin wax. Five- micrometer -thick paraffin sections were mounted on gelatin-coated slide glass and air-dried. The sections were deparaffinized by immersing in xylene three times and rehydrate in decreasing concentration of ethanol.

After a pretreatment with 0.3% Triton X-100-containing PBS (pH 7.2) for 30 min and preincubation with 10% normal donkey serum, the sections were incubated with a rat anti-GP2 monoclonal antibody (1:200 dilution; MBL), rabbit anti-Tnfaip2 antibodies (1:200) (7), goat anti-CD3ε antibodies (1:200; Santa Cruz), rat anti-B220 monoclonal antibody (1:100; Biolegend), goat anti-CCL9 antibodies (1:100; R&D systems), goat anti-CCL20 antibodies (1:200; R&D systems), rabbit anti-RelB antibodies (1:200; Santa Cruz), guinea pig anti-Sox8 antisera (17), or goal anti-RANK antibodies (1:400; R&D systems) at room temperature overnight, followed by incubation with appropriate secondary antibodies for 2 h at room temperature. For nuclear staining, Hoechst 33342 (Life Technologies) was used after incubation with the secondary antibodies. The specimens were observed using a confocal laser microscope FV1000 (Olympus) after mounting with SlowFade® Gold antifade reagent (Life Technologies).

To achieve GP2 staining in the paraffin sections, sections were dipped in 0.1% H_2_O_2_ after the pretreatment with Triton X-100 and donkey serum in order to inhibit endogenous peroxidase activities. The sections were then incubated with a rat anti-GP2 monoclonal antibody (1:200 dilution; MBL) at room temperature overnight. Subsequently, the specimen were further incubated with horseradish peroxidase (HRP)-labeled anti-rat IgG for 2 h. The sites of the antigen-antibody reaction were visualized by 0.01% 3,3′-diaminobenzidine tetrahydrochloride and 0.001% (v/v) H_2_O_2_ in Tris-HCl (pH 7.2). For nuclear staining, hematoxylin was used. The specimens were observe using a research light microscope, BX51 (Olympus), and images were captured with a digital color camera, DP71 (Olympus).

### RANKL Administration

The primers 5′-CACCCCCGGGCAGCGCTTCTCAGGAGCT-3′ and 5′-GAGACTCGAGTCAGTCTATGTCCTGAAC-3′ (Sigma Genosys) were used for polymerase chain reaction (PCR) to amplify a cDNA clone of RANKL. The PCR fragment was subcloned into the pGEX-4T-2 vector (GE Healthcare) after digestion by SmaI and XhoI. The construct was transformed into the BL21 Escherichia coli strain for glutathione-S-transferase (GST) fusion protein expression. The culture was induced with 0.1 mM isopropyl β-D-1-thiogalactopyranoside for 16 h at 20°C, and GST-RANKL was purified from bacterial lysate by affinity chromatography on a Glutathione-Sepharose 4B (GE Healthcare) followed by dialysis against multiple changes of PBS. Recombinant GST used as a control was prepared by the same method using an empty pGEX-4T-2 vector. Purified protein was administered to mice by intraperitoneal injections of 10 mg/Kg per day for 3 days. After 24 h from the last administration, the mice were sacrificed and subjected to the following assays.

### Silver-Intensified Immunogold Method for Transmission Electron Microscopy

PFA-fixed and decalcified tissues were dipped in 30% sucrose solution overnight at 4°C, embedded in O.C.T. compound, and quickly frozen in liquid nitrogen. Fifteen-micrometer-thick frozen sections were mounted on poly-L-lysine coated glass slides, incubated with the rat anti-GP2 antibody (1 μg/ml) overnight, and subsequently reacted with goat anti-rat IgG covalently linked with 1 nm gold particles (1:200 dilution; Nanoprobes). Following silver enhancement with a kit (HQ silver^TM^; Nanoprobes), the sections were osmicated, dehydrated, and directly embedded in Epon (Nisshin EM). Ultrathin sections were prepared and stained with uranyl acetate and lead citrate for observations under an electron microscope (H-7100; Hitachi).

### Quantitative PCR Analysis

Total RNA was prepared using TRIzol® (Life Technologies) from the trachea resected from under the larynx to the bronchial main branches of mice injected with GST or GST-RANKL. First-strand cDNA synthesis was completed using ReverTra Ace® (TOYOBO). Quantitative PCR reactions were conducted in Rotor Gene 6000 equipment (Qiagen) or StepOnePLUS^TM^ (Thermo Fisher Scientific K.K.) using KAPA SYBR® Green Fast PCR kit (KAPA Biosystems). The specific primers used are shown in Supplementary Table 1.

### Treatment of Latex Beads in to Mice

Aliquots of 1 × 10^11^/ml fluorescent polystyrene latex beads (100 nm in diameter; Life Technologies) were dropped into the nasal cavity of C57BL/6N mice administrated with GST or GST-RANKL as described above three times. After a 20-min incubation, mice were sacrificed and subjected to immunohistochemical experiments.

### Fluorescence *in situ* Hybridization

FISH was performed using the Quantigene® View RNA ISH Cell Assay (Affymetrix) with slight modifications in the fixation and protease digestion steps. Briefly, perfusion fixation was performed with a solution containing 4% PFA in PBS. The trachea was removed and immersed in 4% PFA for an additional 24 h. The preparation of frozen sections was described as above. The sections were pretreated with a detergent solution for 10 min and then protease QS (dilution 1:400 in PBS; Affymetrix) or 0.1 mg/ml proteinase K in PBS (Kanto Chemical) for 10 min at room temperature. Subsequent processes were performed in accordance with the manufacturer's protocol. Specific oligonucleotide probe sets against *Tnfrsf11a* (catalog No, VB1-13969) was purchased from Affymetrix, Inc.

### Air-Liquid Interface Culture

Tracheal cells from C57Bl/6N, BALB/c, and ddY mice were used for the culture of tracheal M cells in air-liquid interface (ALI) culture condition, according to a previous report with some modification (21). For most studies, ddY mice, 3–6 week of age were used. Mice were euthanized, and the tracheas were resected from the larynx to the bronchial main branches and collected in ice-cold Dulbecco's modified eagle medium (DMEM) with 10% fetal bovine serum (FBS) and antibiotics-antimycotics, and then transferred into fresh DMEM containing collagenase/dispase. The tracheas were incubated for 18–24 h at 4°C with mixing by gently inverting. The cells were collected by three time wash with DMEM containing 10% FBS and antibiotics-antimycotics. After incubation in Accutase™ (Nalarai) for 30 min at 37°C, the cells were centrifuged at 300 g for 5 min at 4°C, and resuspended in DMEM containing 10% FBS and antibiotics-antimycotics. After incubation in tissue culture plates for 3–4 h in 5% CO2 at 37°C to adhere fibroblasts, non-adherent cells were collected by centrifugation, the unattached cells were resuspended in the airway epithelial cell growth medium (Promocell) with 10 μM Y-27632 (FUJIFILM Wako Pure Chemical Industries) and cultured on collagen-coated Transwell® cell culture inserts (pore size 0.4 μm).

### Immunocytochemistry for ALI Cultured Cells

The cell on the Transwell® membrane were fixed with 4% PFA in PBS for 15 min, washed in PBS, cut from supports into two pieces, and processed for immunofluorescence analysis in 24-well-palate. The cells were incubated with anti-Spi-B antibody, anti-GP2 antibody, anti-Tnfaip2 antibody, or Alexa 647 conjugated phalloidin in 0.2% saponin and 0.2% BSA overnight at 4°C. For nuclear staining, Hoechst 33342 (Life Technologies) was used after incubation with the secondary antibodies. After washing with PBS three times, the cells were incubated with appropriate secondly antibodies for 2 h at room temperature.

### Elastase-Induced Emphysema and Pneumococcal Infection Mouse Model

In the elastase-induced emphysema model, mice were intratracheally injected with 5 U of porcine pancreatic elastase (Elastin Products) (22, 23). In the chronic obstructive pulmonary disease (COPD) exacerbation model, the mice were intranasally inoculated with 1 × 10^7^ colony-forming units (CFUs) of *Streptococcus pneumoniae* serotype 6°C at 4 weeks after elastase injection. Clinical specimens were isolated and suspended in 50 μL of sterile PBS as previously described. The cells were observed using a confocal laser microscope FV1000 (Olympus) after mounting with SlowFade® Gold antifade reagent (Life Technologies).

### Mouse Models of Lung Inflammation Due to Acute and Chronic CS Exposure

Lung inflammation was induced by cigarette smoke (CS) exposure as previously described (22, 24). In brief, mice were exposed to mainstream CS generated from commercially available filtered cigarettes (Marlboro; Philip Morris) (12 mg tar/1.0 mg nicotine). CS inhalation was achieved by using a cigarette smoke exposure system (Model SIS-CS; Shibata Scientific Technology). Mice were exposed to CS for 60 min/day and 5 days/week over 3 months. Mice were killed 3 months after CS exposure. As a control, mice inhaled normal air under the same conditions were used.

### LPS-Induced iBALT Formation Model

iBALT was induced by LPS administration as previously describes (25). In brief, litters of neonatal or adult mice were anesthetized with isofluorane and were given 1–10 μg LPS in a volume of 10 μl (0–2 weeks of age) or 30 μl (2 weeks of age and older) intranasally every other day over 10 d (five doses).

### Quantitative Image Analysis

For quantification of sections, at least 2 histological sections of trachea from each animal were subjected to immunofluorescence analysis and at least 10 images from each section with FV1000 confocal laser scanning microscope (Olympus). The area of trachea epithelia, the number of tnfaip2-positive or negative cells were measured by ImageJ software (26).

### Statistical Analysis

Differences between mean values for two or more groups were analyzed by Student's *t*-test or one-way ANOVA with Tukey-Kramer test, respectively, using Prism (GraphPad Software, La Jolla, CA, USA). All statistical analyzes were performed on at least two independent experimental data.

## Results

### GP2^+^Tnfaip2^+^ Cells in the Tracheal Epithelium of Healthy Mouse

**Figure 1:**
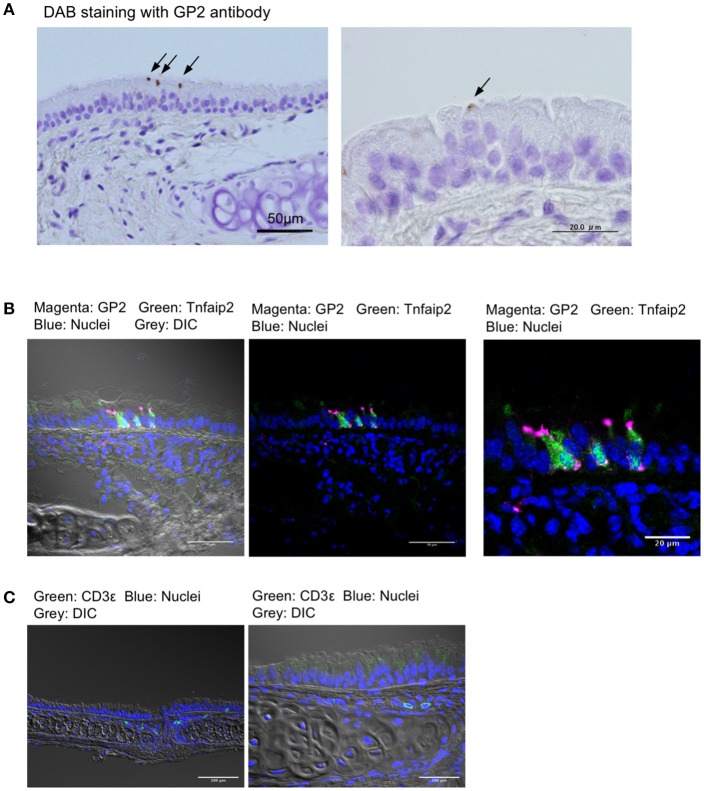
GP2^+^Tnfaip2^+^ cells in the tracheal epithelium. **(A)** Immunohistochemical images of GP2 in the trachea of BALB/c mice. Immunoreactivities of GP2 antibody are visualized with DAB chromogen with H_2_O_2_ (arrows), and tissues are counterstained with hematoxylin. Bars: 50 μm (left panel) and 20 μm (right panel). **(B)** Immunofluorescence images of Tnfaip2 (green) and GP2 (magenta) in the trachea of BALB/c mice. DIC (gray), differential interference contrast; Bars: 50 μm (left panel) and 20 μm (right panel). **(C)** Immunofluorescence images of CD3ε (green). Bars: 100 μm. Nuclei were stained with DAPI (blue) in **(B,C)**.

We first stained the trachea for GP2, a molecular marker for M cells in the intestinal Peyer's patches, as well as nasopharynx-associated lymphoid tissue (NALT) (7, 8, 20). Immunohistochemical experiments using the anti-GP2 monoclonal antibody revealed distinctive positive staining on a small number of cells that were scattered in the pseudostratified epithelium of the trachea (Figure 1A). Immunofluorescence analysis revealed that GP2^+^ cells also reacted with the anti-Tnfaip2 antibody, the antigen of which was previously shown to be selectively expressed in M cells (Figure 1B) (7, 9). At a higher magnification, Tnfaip2 was found throughout the cytoplasm of cells, while GP2 signals occurred on the luminal side of cells and as puncta in the deeper region of the tracheal epithelium (Figures 1A,B). We found a few CD3ε^+^ T lymphocytes in the subepithelial connective tissues of the trachea (Figure 1C), but failed to find B220^+^ B cells.

### RANKL Induces GP2^+^Tnfaip2^+^ Cells in the Tracheal Epithelium

**Figure 2:**
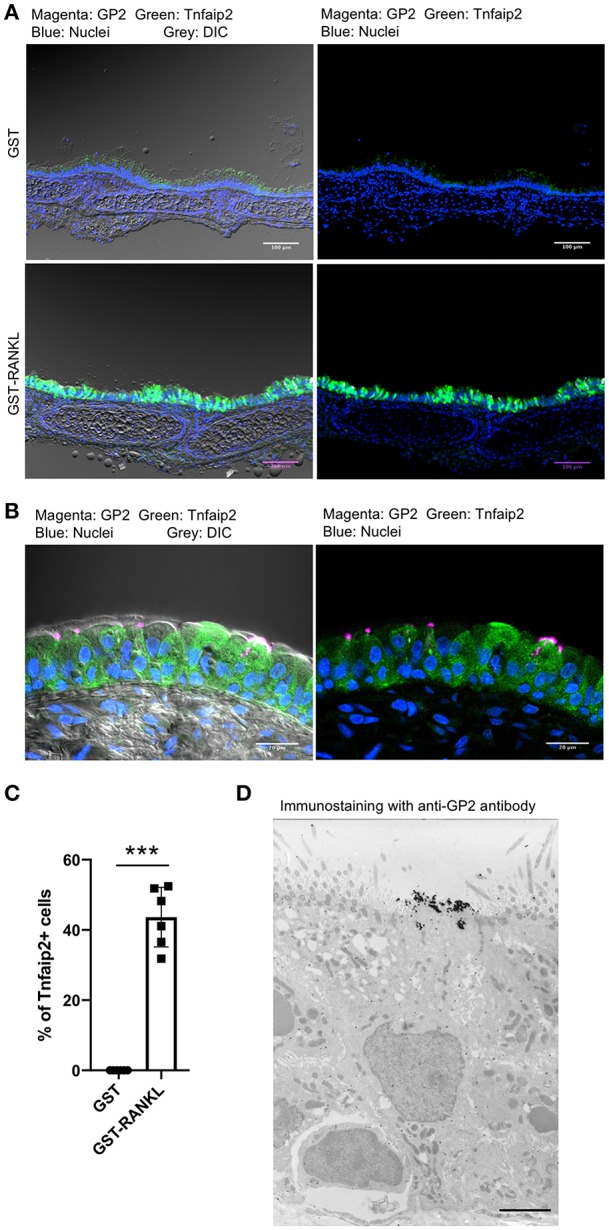
Administration of RANKL into mice induces GP2 and Tnfaip2 expression in the tracheal epithelium. **(A)** Immunofluorescence images of GP2 (magenta) and Tnfaip2 (green) in the trachea of C57BL/6N mice administered 10 mg/kg GST (control) or GST–RANKL daily for 3 days. Blue is nuclei stained with DAPI. DIC, differential interface contrast; Bars: 100 μm. (**B**) A high-magnification image of **(A)**. Bars: 20 μm. **(C)** Quantification of Tnfaip2-positive cells in the trachea. ***P < 0.005 calculated with Student's *t*-test. Data were obtained from 6 mice in each group from 3 independent experiments. The percentage of Tnfaip2-positive cells to epithelial cells was calculated and expressed as mean ± standard deviation. **(D)** A representative immunoelectron microscopic image of GP2 (black dots). Bar: 10 μm.

**Figure 3:**
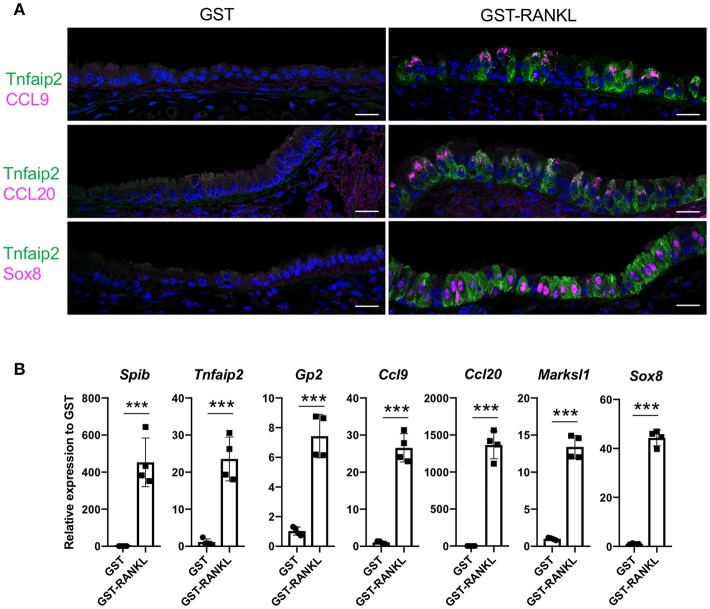
Administration of RANKL into mice induces M-cell-associated gene expression in the trachea. **(A)** Immunofluorescence images of the trachea of C57BL/6N mice administered 10 mg/kg GST (control) or GST–RANKL daily for 3 days. Magenta is CCL9, CCL20, or Sox8 as indicated. Green is Tnfaip2. Blue is nuclei stained with DAPI. Bars: 20 μm. **(B)** Quantitative PCR analysis of the trachea of C57BL/6N mice administered 10 mg/kg GST or GST–RANKL daily for 3 days. Data were obtained from 4 mice in each group from 2 independent experiments and expressed as mean ± standard deviation. ***P < 0.005 (Student's *t*-test).

RANKL has been shown to initiate the development of M cells in the intestine (13). The intraperitoneal injection of recombinant GST-fused RANKL (GST–RANKL) induces ectopic M cells in the villus epithelium of mice administered 10 mg/kg GST–RANKL daily for 3 days (13). In this situation, we found that the number of GP2^+^Tnfaip2^+^ cells was conspicuously increased in the tracheal epithelium (Figures 2A–C). Injections of GST under the same conditions, as a negative control, had no such effect. Notably, 43.65 ± 3.47 cells in the tracheal epithelial cells of C57BL/6N mice were Tnfaip2^+^ cells after the administration of GST–RANKL (Figure 2C). Immunoelectron microscopy revealed that the GP2^+^ cells in the trachea were non-ciliated epithelial cells (Figure 2D). The effects of RANKL on the tracheal epithelial cells were similar among C57BL6/N, BALAB/c, and ddY strains. The Tnfaip2^+^ cells induced by RANKL administration were also positive for other M-cell markers, CCL9, CCL20, and Sox8 (Figure 3A) (7, 17). By performing quantitative PCR analysis, we further confirmed the elevated expression of M-cell-associated genes—*Spib, Tnfaip2, Gp2, Ccl9, Ccl20*, and *Marksl1*—after the administration of RANKL in the trachea (Figure 3B). The lower respiratory tract is composed of the larynx and the trachea; the trachea branches stepwise with the bronchus and bronchioles, and finally continues to the respiratory bronchioles with alveoli. The GP2^+^Tnfaip2^+^ cells appeared in the pseudostratified epithelium continuously from the larynx to the respiratory bronchioles (Supplementary Figure 1). The administration of GST–RANKL did not induce any Tnfaip2^+^GP2^+^ cells on the stratified squamous epithelia of the pharynx or on the alveolar epithelium (Supplementary Figure 1). The tracheal glands were immunopositive for GP2 in the presence or absence of RANKL, but negative for Tnfaip2 (Supplementary Figure 1). This is consistent with our previous reports revealing that several mucous glands express and secrete GP2 (27, 28).

### GP2^+^Tnfaip2^+^ Cells Have High Uptake Capacity

**Figure 4:**
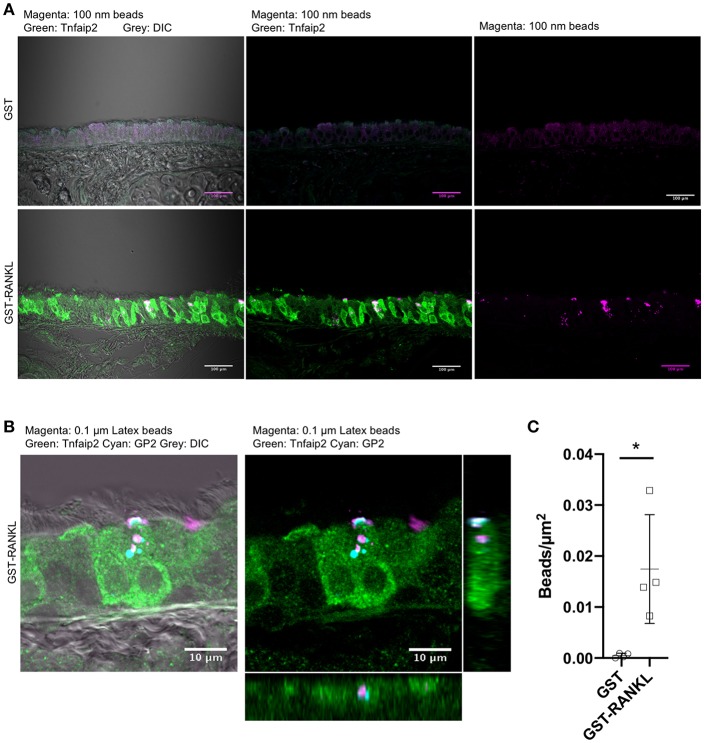
Uptake of luminal latex beads by GP2^+^Tnfaip2^+^ cells in the trachea. **(A)** Latex beads (magenta) were dropped into the nasal cavity of C57BL/6N mice administered with GST or GST-RANKL as described in the method section. Immunofluorescence images of the trachea immunostained for Tnfaip2 (green). Bars: 100 μm. **(B)** Confocal images of the tracheal epithelium immunostained for Tnfaip2 (green) and GP2 (cyan). Orthogonal images from confocal images. **(C)** Quantification of the number of latex beads in the tracheal epithelia. Data expressed as mean ± standard deviation of the number of beads relative to the area of the epithelia (beads/μm^2^) were shown. Data were obtained from 4 mice in each group from 2 independent experiments. *P < 0.05 calculated with Student's *t*-test.

To confirm that GP2^+^Tnfaip2^+^ cells in the lower respiratory tract were functionally capable of taking up particles from the lumen, we administered fluorescent microbeads into the nasal cavity of animals administered GST–RANKL or GST. Confocal microscopic observations showed that microbeads were incorporated by Tnfaip2^+^ cells of the trachea of GST–RANKL-injected mice (Figures 4A,B). GP2 was localized inside cells with a punctate pattern, and some of the dot-like immunoreactivities for GP2 were colocalized with incorporated microbeads, indicating that GP2 at the apical cell surface was endocytosed together with microbeads (Figure 4B). Statistical analysis confirmed that the number of microbeads incorporated into the tracheal epithelium was significantly increased in the animals injected with GST–RANKL (Figure 4C). In this experimental condition, however, we could not detect any microbeads in the sub-epithelial regions containing tracheal lamina propria, the draining lymph nodes, and blood vessels.

### Low but Sufficient Expression of RANK in the Tracheal Epithelium

We next investigated the expression of RANK, a receptor of RANKL. Immunofluorescence analysis with RANK antibodies showed a small number of RANK-positive cells in the tracheal epithelium under normal conditions (Supplementary Figure 2A). The RANK-positive cells also reacted with anti-GP2 antibody: Immunoreactivities of RANK were never detected in GP2-negative cells (Supplementary Figure 2A). Interestingly, RANKL administration significantly increased the expression level of *Tnfrsf11a*, which encodes RANK mRNA, in the trachea (Supplementary Figure 2B). Accordingly, the number of RANK-immunoreactive cells was increased by RANKL (Supplementary Figure 2C). Given that RANKL administration induced GP2^+^ M cells, RANK-positive cells may be differentiated and matured M cells.

**Figure 5:**
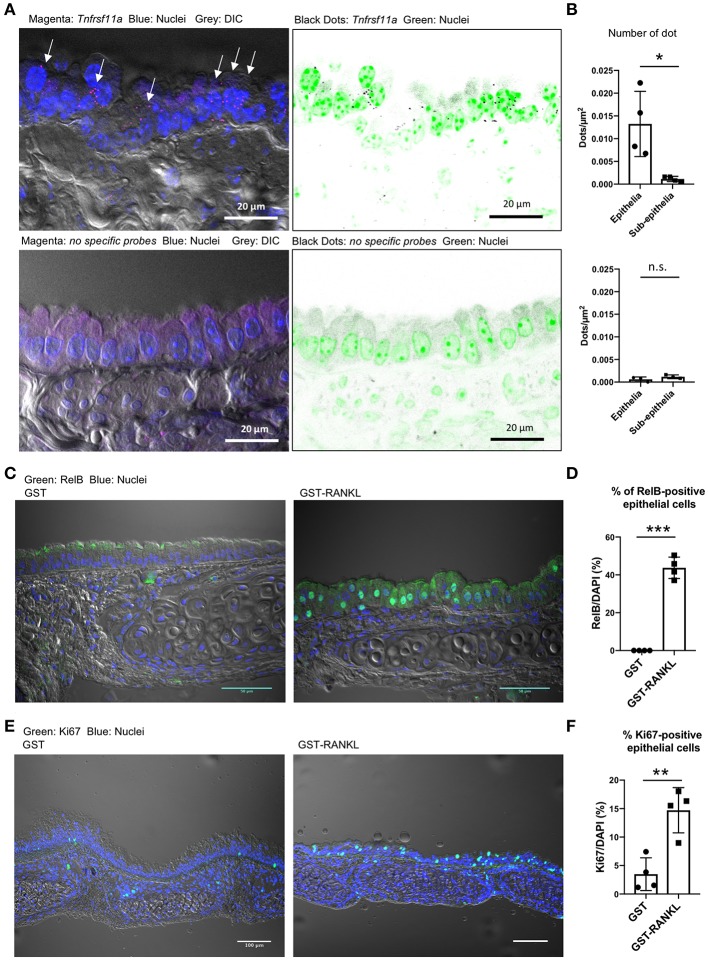
Receptor activator of NFκB (RANK) expression in the tracheal epithelium. **(A)** Fluorescence *in situ* hybridization (FISH) images of the tracheal epithelium of C57BL/6N mice using oligonucleotide probes for *Tnfrsf11a* encoding RANK (magenta dots in the left panels and black dots in the right panels). Arrows in the left panel indicate Tnfrsf11a-positive cells. Nuclei are colored with blue in the left panels and green in the right panels. Bars: 20 μm. **(B)** Quantification of FISH signals in the epithelial and subepithelial regions. Lower panels show FISH images using non-specific probes as a negative control. *P < 0.05, n.s., not significant calculated by Student's *t*-test. Data was obtained from 4 mice in each group from two independent experiments. The number of dots relative to the area of the epithelia or sub-epithelia (dots/μm^2^) was shown. Data expressed as mean ± standard deviation. **(C,E)** Administration of RANKL induces the accumulation of RelB (**C**: green) in the nuclei of the tracheal epithelium, and expands Ki67-positive cells (**E**: green) there. C57BL/6N mice were intraperitoneally injected with GST or GST–RANKL. After 24 h, mice were sacrificed and the trachea was extracted for immunofluorescence analysis. Representative immunofluorescence images of RelB are shown. Bars: 50 μm in **(C)** and 100 μm in **(E)**. **(D,F)** Quantification of RelB-positive cells **(D)** and Ki67-positive cells **(F)**. ***P < 0.005 and **P < 0.01, calculated with Student's *t*-test. Data were obtained from 4 mice in each group from 2 independent experiments in **(D)**, and 4 mice in each group from 3 independent experiments in **(F)**. Data expressed as mean ± standard deviation.

We performed single-molecule fluorescence *in situ* hybridization (FISH) and showed that *Tnfrsf11a* was expressed broadly in the tracheal epithelial cells under normal conditions (Figure 5A). Specific signals of the oligoprobes were detected in the epithelium as bright dots, whereas similar positive signals were hardly detected in the tissue sections treated with non-specific oligoprobes (Figure 5A). Indeed, the quantification of FISH signals revealed that the number of dots were significantly greater in the epithelium than in the subepithelial connective tissue (Figure 5B). Activation of RelB, a transcription factor downstream of RANKL–RANK signaling, is essential for initiating M-cell differentiation (7, 29). We detected the nuclear translocation of RelB in 43.74 ± 2.899% (mean ± standard error) of the tracheal epithelium at 24 h after RANKL administration (Figures 5C,D). Ki-67 staining showed that the administration of GST–RANKL induced cell proliferation in the epithelium (Figures 5E,F). These findings indicate that RANK is expressed broadly in the tracheal epithelium at a low level, but this may be sufficient for inducing downstream RelB activation.

### Two-Dimensional Culture of Airway M Cells

**Figure 6:**
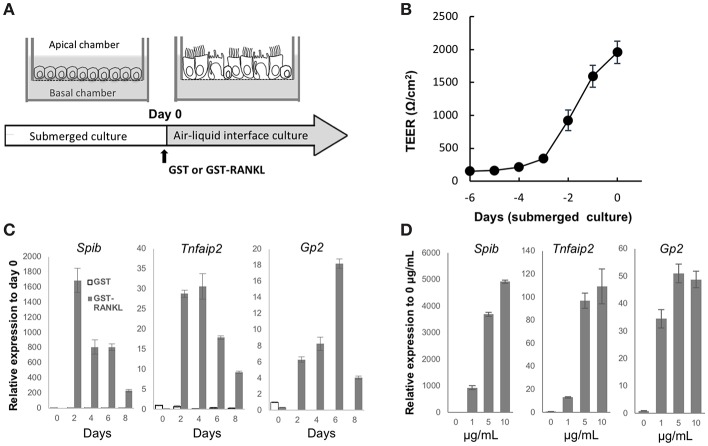
RANKL induces expression of M-cell associated genes in air-liquid interface culture of tracheal epithelial cells. **(A)** Schematic diagram depicting the air–liquid interface (ALI) culturing protocol. Tracheal epithelial cells harvested from ddY mice were cultured on Transwell culture inserts under submerged conditions. After the cells became confluent, they were cultured under ALI conditions with 1 μg/mL GST or GST–RANKL. **(B)** Representative graph of the change of transepithelial electric resistance during submerged culture. **(C)** Time course of the expression of M-cell-associated genes in the ALI culture of tracheal epithelial cells that were harvested every other day after GST or GST–RANKL administration for 8 days. **(D)** Dose dependence of GST–RANKL for the induction of M-cell-associated genes in the tracheal epithelial cells. Cells were cultured for 6 days under ALI conditions with the indicated concentration of GST–RANKL. Representative data of three individual experiments are shown in **(B–D)**. The bars on the graph represent the standard deviation of triplicate samples.

Air–liquid interface (ALI) cultures of mouse tracheal epithelial cells are a well-established model to study the differentiation and function of airway epithelial cells (21). To obtain a deeper understanding of airway M cells, we used ALI culture of the tracheal epithelium (Figure 6A). Isolated epithelial cells were cultured under submerged conditions until they established a confluent monolayer, as revealed by monitoring transepithelial electrical resistance (Figure 6B). Subsequently, cells were transferred to ALI conditions and cultured with GST, as a control, or GST–RANKL. Quantitative PCR analysis showed that *Spib*, which is an essential transcription factor for initiating M-cell differentiation, was elevated within 2 days after RANKL treatment (Figure 6C). *Tnfaip2* expression was also increased within 2 days and peaked after 2–4 days (Figure 6C). The expression of *Gp2*, which is a marker of mature M cells, was expanded and reached its peak after 6 days (Figure 6C). The expression levels of M-cell associated genes were increased in a manner dependent on the GST–RANKL concentration in the ALI culture (Figure 6D).

**Figure 7:**
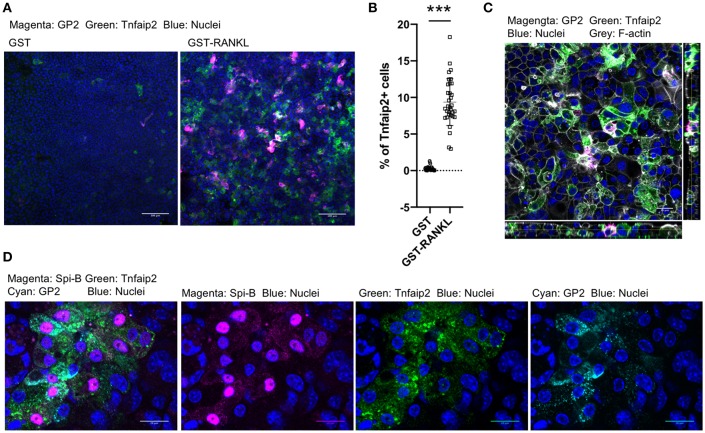
RANKL induced GP2^+^Tnfaip2^+^ cells in the tracheal epithelial culture. **(A)** Harvested tracheal epithelial cells were cultured with 5 μg/mL GST or GST–RANKL for 6 days under ALI conditions. Immunofluorescence images of GP2 (magenta) and Tnfaip2 (green) are shown. Nuclei were stained with DAPI (blue). Bars: 100 μm. **(B)** Quantification of the area of Tnfaip2-positive cells. Data were obtained from 33 area in each group from 3 independent experiments and expressed as mean ± standard deviation. ***P < 0.005 calculated with Student's *t*-test; **(C)** Orthogonal images of Tnfaip2 (green), GP2 (magenta), F-actin (gray), and nuclei (blue) from confocal laser scanning microscopy. Bar: 20 μm. **(D)** Immunofluorescence images of Spi-B (magenta), Tnfaip2 (green), and GP2 (cyan). Nuclei were stained with DAPI (blue). Bars: 20 μm. Images are representative of at least three independent experiments.

**Figure 8:**
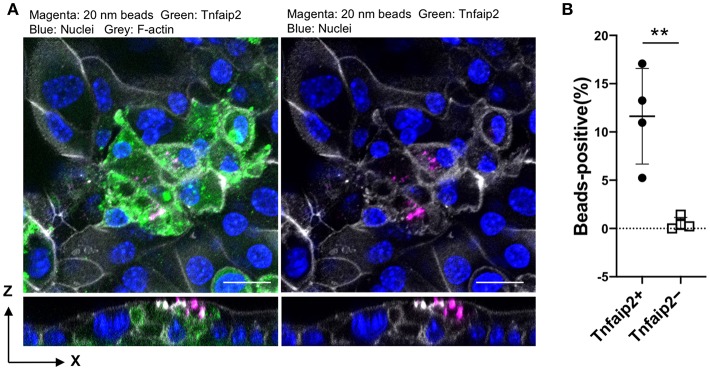
Uptake of nanobeads by airway M cells cultured under ALI conditions. **(A)** Tracheal epithelial cells were cultured with 5 μg/mL GST–RANKL for 6 days under ALI conditions. On the sixth day, culture medium containing 20-nm fluorescent nanobeads was added to the upper chamber. One hour later, the culture medium for the cells was changed to fresh complete medium, followed by additional culture for 1 h. After that, the cells were fixed and stained for Tnfaip2 and F-actin. Confocal fluorescence images of Tnfaip2 (green), F-actin (gray), and nanobeads (magenta) are shown. Bars: 20 μm. **(B)** Quantification of nanobeads in the Tnfaip2-positive cells. The percentage of beads-positive cells were measured. And Data expressed as mean ± standard deviation. **P < 0.01 (Student's *t*-test) Data were obtained from 4 areas of confocal 3D image from two independent experiments.

We further confirmed the induction of tracheal M cells in the ALI culture by immunofluorescence analysis. The GP2^+^Tnfaip2^+^ cells were induced in the ALI culture in the presence of GST–RANKL (Figure 7A). Quantitative analysis revealed that the percentage of Tnfaip2^+^ cells was approximately 9.35 ± 3.2% (mean ± standard deviation) in the cells treated with GST–RANKL (Figure 7B). The cells were confirmed to reside in a single layer by the three-dimensional reconstitution of confocal images with F-actin staining (Figure 7C). The triple staining revealed that GP2^+^Tnfaip2^+^ cells expressed Spi-B (Figure 7D). The Tnfaip2^+^ cells had significantly higher ability to take up 20-nm beads added into the apical medium than the Tnfaip2^−^ cells (Figures 8A,B). These findings revealed that RANKL induced functional M cells in the ALI culture of isolated tracheal epithelial cells.

### Pathological Induction of Airway M Cells in Murine Models of Respiratory Disease

**Figure 9:**
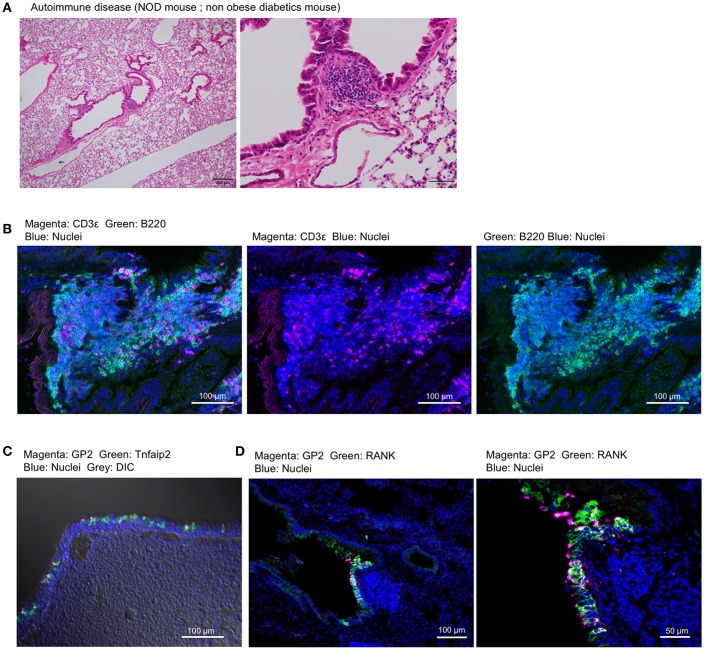
Airway M cells are pathologically induced in the tracheal epithelia associated with lymphoid infiltration in murine models of autoimmune disease. **(A)** Hematoxylin–eosin staining of the lung of non-obese diabetic (NOD) mice. Bars: 200 μm (left panel) and 50 μm (right panel). **(B)** Immunofluorescence images of CD3ε (magenta) and B220 (green) of lymphoid follicles associated with the bronchiole of NOD mice. Bars: 100 μm. **(C,D)** Immunofluorescence images of GP2 (magenta) and Tnfaip2 (green) **(C)** as well as GP2 (magenta) and RANK (green) **(D)** in the trachea of NOD mice. Images are representative of three independent experiments.

Non-obese diabetes (NOD) mice spontaneously develop type 1 diabetes and autoimmunity affecting multiple organs (30). We found the lymphoid follicles near the trachea or bronchioles of NOD mice by histological analysis (Figure 9A). The lymphoid follicles consisted primarily of B220^+^ B cells and a relatively small number of CD3ε^+^ T cells with no clear T-cell zone (Figure 9B). GP2^+^ M cells, which express Tnfaip2 and RANK, appeared in the epithelium associated with lymphoid follicles (Figures 9C,D).

**Figure 10:**
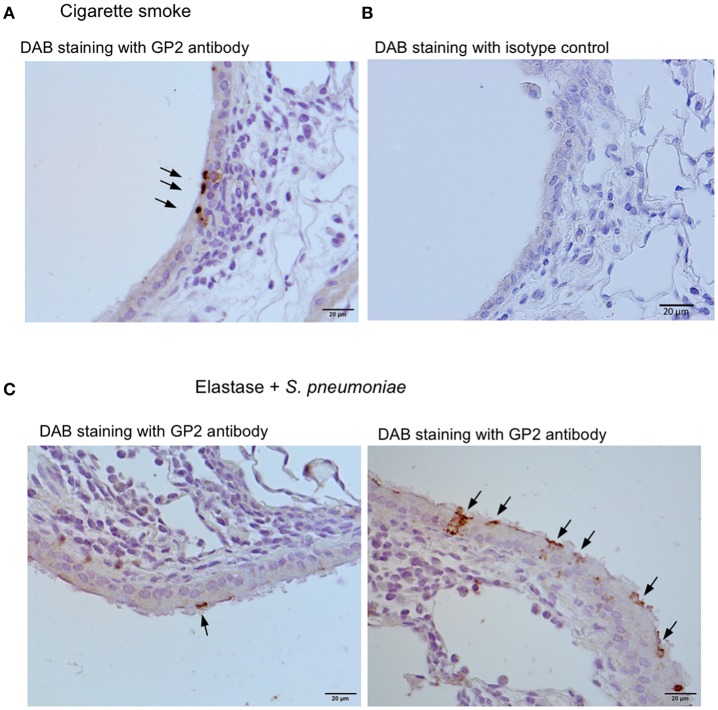
Airway M cells are pathologically induced in the tracheal epithelia associated with lymphoid infiltration in murine models of respiratory disease. Immunohistochemical images of GP2 (arrows) in the trachea of the mice that inhaled cigarette smoke **(A)** or elastase with *S. pneumonia* **(C)**. Five mice of each disease model and (5 sections per an animal) were observed, and representative images were shown. **(B)** The section of the trachea of the mice inhaled cigarette smoke was treated with an isotype control (Rat IgG2a) of anti-GP2 antibody for the negative control of immunohistochemistry. Bars: 20 μm.

We further investigated three murine models of respiratory disease. Long-term exposure of mice to cigarette smoke was reported to cause lung inflammation (22, 24). Moreover, the intratracheal injection of elastase into mice induced emphysema, and subsequent infection of *S. pneumoniae* aggravated the symptoms of emphysema including lymphocytic infiltration (22, 23). Lipopolysaccharide (LPS)-induced inflammation in the respiratory tract in neonate mice was shown to cause the ectopic formation of lymphoid follicles (25). In these experimental models, we frequently found GP2^+^ cells or Spi-B^+^ cells in the tracheal epithelium associated with lymphocytic infiltration (Figure 10).

## Discussion

Here, we characterized M cells, which initiate immune responses to luminal materials, in the lower airway. RANKL effectively induced the airway M cells in the murine trachea, which express known M-cell marker proteins of the intestine—GP2, Tnfaip2, Ccl9, Ccl20, and Sox8—and have a high capacity to take up nanobeads from their apical surface. Based on this finding, we further established a two-dimensional culture system for the airway M cells. Recent studies have established the *in vitro* generation of intestinal M cells by using enteroid culture from intestinal Lgr5^+^ stem cells (14, 29, 31). This has contributed to revealing the differentiation process of M cells. For the functional analysis of intestinal epithelium, several groups have further developed methods to grow the enteroid in a two-dimensional culture, which is also applicable to M cells, using cell culture inserts that separate luminal and subepithelial compartments (32, 33). On the other hand, the molecular characteristics, differentiation mechanisms, and function of airway M cells have remained largely unknown. To our knowledge, the current study is the first to report on a two-dimensional culture system of airway M cells, which was developed based on the well-established culture system of tracheal epithelial cells. Airway M cells can mediate respiratory infection by bacteria such as *Streptococcus pyogenes* and *Mycobacterium tuberculosis* (34, 35). The airway M cell culture system, therefore, should be useful for future experiments to clarifying the physiological and pathological significance of airway M cells, and to clarify differences between them and intestinal M cells.

M cells are generally associated with underlying lymphoid follicles that are, however, rarely formed in the trachea of healthy human and mouse. Indeed, we detected only a few T cells, but no B cells, in the subepithelial connective tissues of the murine trachea, and did not detect any topographical association of M cells with these T cells. Inducible bronchus-associated lymphoid tissue (iBALT) is an ectopic lymphoid tissue that forms in the lungs following pulmonary inflammation or infection (36). The leukocytes comprising iBALT are arranged in the B-cell follicle and the T-cell zone, in a way that resembles the organization of conventional secondary lymphoid organs. On the other hand, iBALT does not have the archetypal FAE as well as subepithelial dome region where dendritic cells accumulate and internalize translocated antigens. Although antigen-transporting M cells have been reported in the iBALT of some species based on their morphological features and the findings of lectin histochemistry (37–39), M cells are considered not to be an essential component of iBALT because they are not consistently observed (36). In the present study, we found in M cells by using molecular markers in four different mouse models of iBALT formation. The mature M cells appeared in the iBALT-associated epithelium under spontaneous autoimmune conditions in the NOD mice. The airway M cells in this disease model highly expressed GP2, Tnfaip2, and RANK, which were shared by both inherent M cells in the trachea of healthy mice and with M cells induced by the administration of GST-RANKL. M cells marked with GP2 or Spi-B were observed in pathologically induced elastase-induced and tobacco smoking-induced emphysema models or in an LPS-induced pulmonary inflammation model, respectively. Our study suggests that the M cells may be a fundamental element constituting iBALT.

M-cell-dependent antigen transcytosis in the intestine plays a key role in the induction of mucosal immune responses to certain antigens. Indeed, we previously showed that the absence of M cells or their antigen uptake receptor GP2 attenuates antigen-specific T-cell responses in mice orally infected with *Salmonella enterica* serovar Typhimurium due to a decrease in bacterial uptake by Peyer's patches (8, 16). Analogously, dysfunction of transcytosis due to the absence of Allograft inflammatory factor 1 (Aif1), which is an actin- and calcium-binding protein highly expressed in intestinal M cells, reduces the uptake of *Yersinia enterocolitica* in Peyer's patches (40). These defects in M-cell-dependent antigen uptake have been shown to eventually diminish the production of antigen-specific secretory IgA (S-IgA) in the gut (8, 41). M cells residing in the iBALT may play a pivotal role in mucosal immune responses at the airway under pathogenic conditions.

The tracheal epithelium mainly consists of ciliated, basal, club, tuft, and goblet cells. We identified M cells as one of the tracheal epithelial cell lineages that specialize in the uptake of luminal materials and are possibly associated with respiratory diseases. The administration of GST–RANKL effectively induced the production of M cells in the trachea as well as the intestine, suggesting that RANKL is a common inducer of M-cell differentiation in these tissues. On the other hand, we failed to detect M cells after RANKL administration in the esophagus, the oral cavity, the urinary tract, or the upper respiratory tract, except the FAE of nasopharynx-associated lymphoid tissue (20, data not shown). Mucosal tissues where M cells are inducible in response to RANKL may be limited and strictly controlled.

RANKL interacts with its receptor, RANK, and activates canonical and non-canonical NFκB pathways via TRAF6 to initiate M-cell differentiation (7, 29). The expression level of RANK in the trachea was, however, lower than in the gut, in which epithelium constitutively expresses RANK. Nevertheless, the rapid nuclear translocation of RelB, non-canonical NFκB, was observed in nearly half of the tracheal epithelial cells by RANKL administration, suggesting that the low-level RANK expression is sufficient for initiating M-cell differentiation. Interestingly, GP2^+^ matured M cells in the lower airway highly express RANK. This suggests the possibility that RANKL administration upregulates RANK expression in the progenitor of M cells, and that RANKL–RANK signaling may also be required for promoting and/or maintaining M cells in the trachea.

Our study revealed that M cells were raised in the tracheal epithelial cells by RANKL stimulation accompanied by cell proliferation. Cell turnover in the adult trachea is generally very low in mammalian airway (42); however, epithelial injury elicits the rapid proliferation of stem/progenitor cells and the tissue is soon repaired. The basal cells maintain a multipotent capacity to differentiate into all types of tracheal epithelial cells (43, 44). Additionally, club cells, non-ciliated secretory cells, also act as stem/progenitor cells to differentiate into ciliated and goblet cells during the wound healing process (45). By focusing on the expression of RANK and the responsiveness to RANKL, it should become possible to clarify the identity of M-cell precursor cells in the respiratory tract.

## Data Availability

All datasets generated for this study are included in the manuscript and/or the Supplementary Files.

## Ethics Statement

All experiments using animals were performed under protocols following the Guidelines for Animal Experimentation, of Hokkaido University Graduate School of Medicine (Protocols No. 15-0139 and No. 18-0034) and the Animal Use Committee at the Keio University School of Medicine approved all animal experiments (protocols No. 12109 and No. 12110).

## Author Contributions

SK: conceptualization and writing–original draft. SK, MM, MH, HS, ST, and YN: methodology and investigation. TA and MI: resources. SK, TI, and KH: writing–review and editing. SK, KH, JI, and TI: supervision. SK and MM: funding acquisition.

## Contribution to the Field Statement

Respiratory diseases caused by infection and allergies have high mortality. In addition to mucociliary and phagocytic clearance systems, there is a large amount of secretory IgA (sIgA), which plays a major role in mucosal defense, in the lower respiratory tract. The mechanism of antigen uptake from the mucosal surface in the lower respiratory tract is not well understood. Therefore, we identified M cells in the lower airway of mice in this study. Given that sampling by M cells is the principle pathway initiating sIgA production in the intestine, our findings should promote the understanding of antigen sampling mechanisms in the airway, as well as of their biological and physiological significance in the respiratory tract. Furthermore, the two-dimensional culture system of airway M cells developed newly in this study should contribute to the further functional studies of M cells in the respiratory immune systems.

### Conflict of Interest Statement

The authors declare that the research was conducted in the absence of any commercial or financial relationships that could be construed as a potential conflict of interest.

## Supplementary Material

Supplementary Figure 1 GP2^+^Tnfaip2^+^ cells appear from the larynx to respiratory bronchiole after RANKL administration. Immunofluorescence images of GP2 (magenta) and Tnfaip2 (green) in the indicated region of C57BL/6N mice administered 10 mg/kg GST or GST–RANKL daily for 3 days. Bars: 20 μm.

Supplementary Figure 2 RANKL enhances RANK expression in the mucosal epithelia. **(A)** Immunofluorescence images of RANK in the trachea from BALB/c mice in a steady state. Bars: 50 μm. **(B)** Quantitative PCR analysis of Tnfrsf11a encoding RANK in the gut epithelia (gut) and the trachea (trachea) of C57BL/6N mice administered 10 mg/kg GST (a negative control) or GST–RANKL daily for 3 days. ^**^*P* <0.01, ^***^*P* <0.005 calculated by the Tukey–Kramer test (4 mice in each experimental condition from two independent experiments). **(C)** Representative immunofluorescence images of RANK (green) in the trachea of C57BL/6N mice treated similarly. Nuclei were stained with DAPI. Bars: 50 μm.

Supplementary Table 1 List of the oligonucleotide primers used for Q-PCR.
