# Supplementary figures and images for: Airway M Cells Arise in the Lower Airway Due to RANKL Signaling and Reside in the Bronchiolar Epithelium Associated With iBALT in Murine Models of Respiratory Disease
Source: Front Immunol. 2019 Jun 11;10:1323. doi: 10.3389/fimmu.2019.01323 (PMC6579949; doi:10.3389/fimmu.2019.01323)

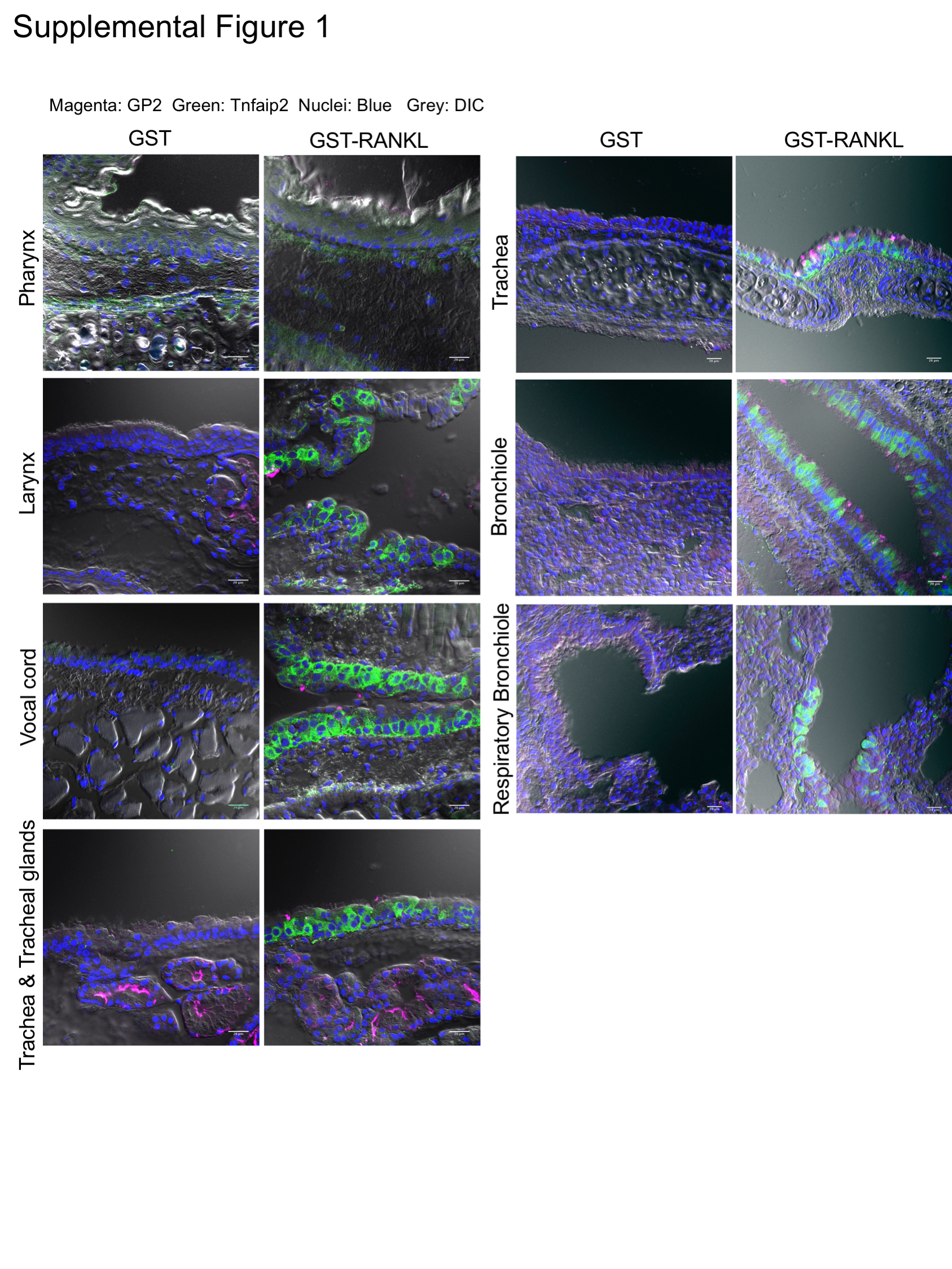

Supplement: Supplementary Figure 1 — GP2+Tnfaip2+ cells appear from the larynx to respiratory bronchiole after RANKL administration. Immunofluorescence images of GP2 (magenta) and Tnfaip2 (green) in the indicated region of C57BL/6N mice administered 10 mg/kg GST or GST–RANKL daily for 3 days. Bars: 20 μm. [file Image_1.TIFF]

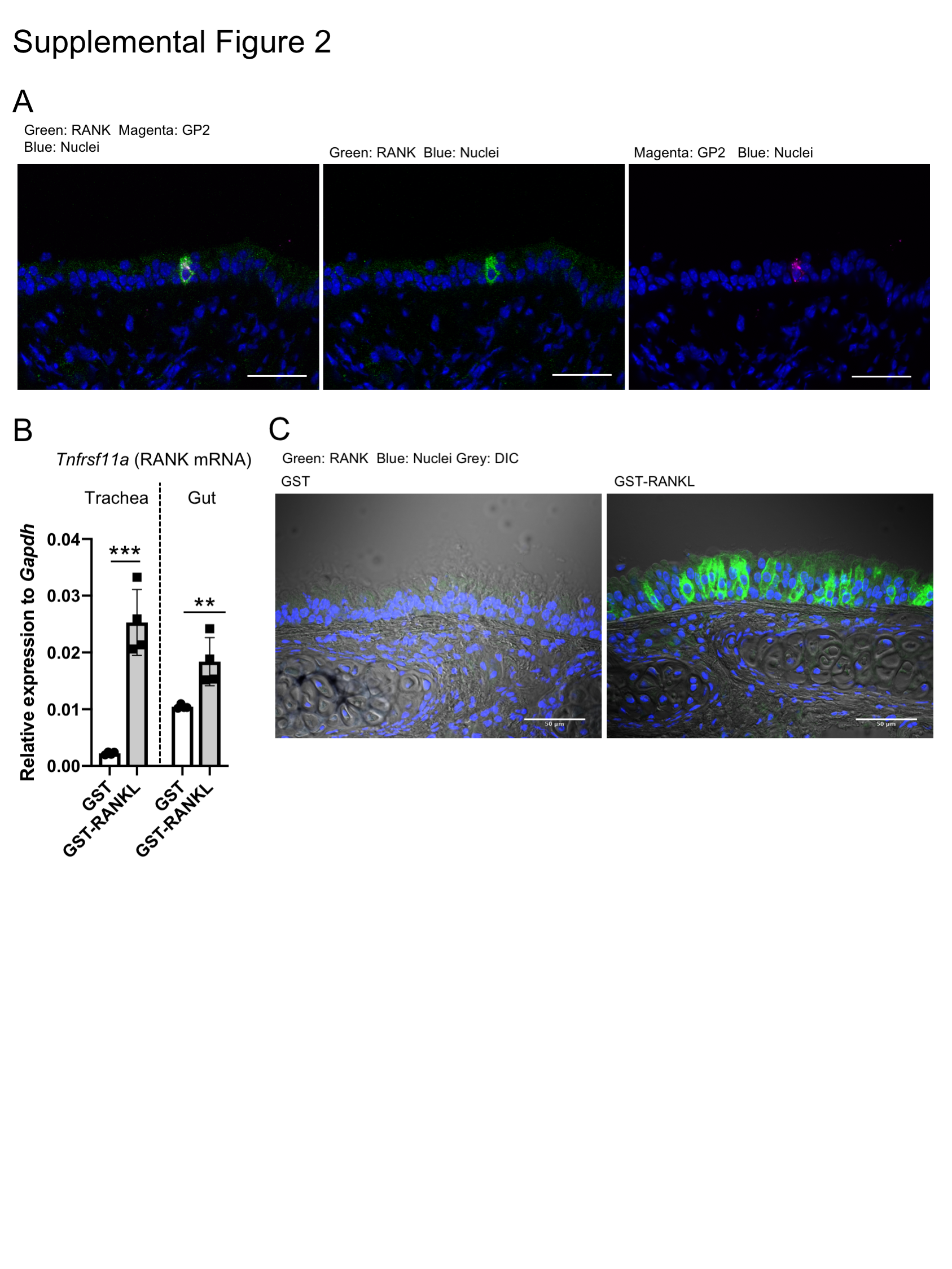

Supplement: Supplementary Figure 2 — RANKL enhances RANK expression in the mucosal epithelia. (A) Immunofluorescence images of RANK in the trachea from BALB/c mice in a steady state. Bars: 50 μm. (B) Quantitative PCR analysis of Tnfrsf11a encoding RANK in the gut epithelia (gut) and the trachea (trachea) of C57BL/6N mice administered 10 mg/kg GST (a negative control) or GST–RANKL daily for 3 days. **P <0.01, ***P <0.005 calculated by the Tukey–Kramer test (4 mice in each experimental condition from two independent experiments). (C) Representative immunofluorescence images of RANK (green) in the trachea of C57BL/6N mice treated similarly. Nuclei were stained with DAPI. Bars: 50 μm. [file Image_2.TIFF]
