# Supplementary material for: Airway M Cells Arise in the Lower Airway Due to RANKL Signaling and Reside in the Bronchiolar Epithelium Associated With iBALT in Murine Models of Respiratory Disease
Source: Front Immunol. 2019 Jun 11;10:1323. doi: 10.3389/fimmu.2019.01323 (PMC6579949; doi:10.3389/fimmu.2019.01323)
Supplement: Supplementary Table 1 — List of the oligonucleotide primers used for Q-PCR. [file Table_1.docx]

| Table S1 | | |
| --- | --- | --- |
| List of the oligonucleotide primers used for Q-PCR | | |
| Target gene (Accession No.) | Forward | Reverse |
| *Sox8* (NM_011447) | TGAATGCCTTCATGGTGTGGG | TTTCACTCAGCAAGCGCCAC |
| *Gp2* (NM_025989) | GTGTGTCCTCAGAAAGCCGA | TGGCTGGTCTACTACTGCGA |
| *Spib* (NM_019866) | GCAAGCGCATGACGTATCAG | GCGTTTGACCTTGCGGATTT |
| *Tnfaip2* (NM_009396) | GTGCAGAACCTCTACCCCAATG | TGGAGAATGTCGATGGCCA |
| *Ccl9* (NM_011338) | GCCCAGATCACACATGCAAC | AGGACAGGCAGCAATCTGAA |
| *Ccl20* (NM_001159738) | TGTACGAGAGGCAACAGTCG | TCTGCTCTTCCTTGCTTTGG |
| *Marcksl1* (NM_010807) | CCCGTGAACGGAACAGATGA | CCCACCCTCCTTCCGATTTC |
| *Gapdh* (NM_008084) | TGTGTCCGTCGTGGATCTGA | TTGCTGTTGAAGTCGCAGGAG |
